# Genetic determinism of spontaneous masculinisation in XX female rainbow trout: new insights using medium throughput genotyping and whole-genome sequencing

**DOI:** 10.1038/s41598-020-74757-8

**Published:** 2020-10-19

**Authors:** Clémence Fraslin, Florence Phocas, Anastasia Bestin, Mathieu Charles, Maria Bernard, Francine Krieg, Nicolas Dechamp, Céline Ciobotaru, Chris Hozé, Florent Petitprez, Marine Milhes, Jérôme Lluch, Olivier Bouchez, Charles Poncet, Philippe Hocdé, Pierrick Haffray, Yann Guiguen, Edwige Quillet

**Affiliations:** 1 Université Paris-Saclay, INRAE, AgroParisTech, GABI, 78350 Jouy-en-Josas, France; 2 SYSAAF, Station LPGP/INRAE, Campus de Beaulieu, 350002 Rennes, France; 3 INRAE, SIGENAE, 78350 Jouy-en-Josas, France; 4 Allice, MNE, 149 rue de Bercy, 75595 Paris, France; 5 Programme Cartes d’Identité des Tumeurs, Ligue Nationale Contre le Cancer, 75013 Paris, France; 6 INRAE, US 1426, GeT-PlaGe, 31326 Castenet-Tolosan, France; 7 INRAE, UMR1095, Gentyane, 63000 Clermont-Ferrand, France; 8 Charles MURGAT Pisciculture, 38270 Beaufort, France; 9 INRAE, LPGP, 35000 Rennes, France; 10 Present Address: The Roslin Institute and Royal (Dick) School of Veterinary Studies, The University of Edinburgh, Midlothian, UK

**Keywords:** Genome-wide association studies, Animal breeding, Genomics

## Abstract

Rainbow trout has a male heterogametic (XY) sex determination system controlled by a major sex-determining gene, *sdY*. Unexpectedly, a few phenotypically masculinised fish are regularly observed in all-female farmed trout stocks. To better understand the genetic determinism underlying spontaneous maleness in XX-rainbow trout, we recorded the phenotypic sex of 20,210 XX-rainbow trout from a French farm population at 10 and 15 months post-hatching. The overall masculinisation rate was 1.45%. We performed two genome-wide association studies (GWAS) on a subsample of 1139 individuals classified as females, intersex or males using either medium-throughput genotyping (31,811 SNPs) or whole-genome sequencing (WGS, 8.7 million SNPs). The genomic heritability of maleness ranged between 0.48 and 0.62 depending on the method and the number of SNPs used for the estimation. At the 31K SNPs level, we detected four QTL on three chromosomes (Omy1, Omy12 and Omy20). Using WGS information, we narrowed down the positions of the two QTL detected on Omy1 to 96 kb and 347 kb respectively, with the second QTL explaining up to 14% of the total genetic variance of maleness. Within this QTL, we detected three putative candidate genes, *fgfa8*, *cyp17a1* and an uncharacterised protein (LOC110527930), which might be involved in spontaneous maleness of XX-female rainbow trout.

## Introduction

The discovery of factors underlying sex determination in fish is a challenge for fundamental biological and evolutionary perspectives and for aquaculture purposes as in some species, managing the sex ratios of farmed stocks is essential for the production efficiency. Unlike birds and mammals which have highly conserved and simple heterogametic genetic sex determination systems, teleost fish species exhibit an amazing diversity of sex determination systems, with different master sex determining genes as well as many minor genetic determinants interacting with environmental effects (in particular temperature) and epigenetic mechanisms^1–3^. Among teleost fish, salmonids are known as strict gonochoric species with the individual sex determined at fertilization and remaining the same throughout their live^1^. In rainbow trout (*Oncorhynchus mykiss*), the genetic determinism of sex has first been described as a male heterogametic system (males XY, females XX) based on the observations of sex-ratios in progenies of hormonally sex-reversed individuals^4,5^ and on the absence of males in gynogenetic offspring^6^. The master gene controlling sex determination in salmonids^7,8^ is an unusual sex determining gene called *sdY* (sexually dimorphic Y chromosome) that evolved from the duplication of the *irf9* (interferon regulatory factor 9) immune related gene. *sdY* is expressed during early gonad differentiation in the testis where it interacts with the conserved female differentiation factor Foxl2^9^ to prevent *cyp19a1a* up-regulation and the subsequent oestrogen production needed for ovarian differentiation^10^. However, despite a strict linkage between the phenotypic sex and the *sdY* locus in many salmonid species^8^, spontaneous masculinisation of XX females have been reported in rainbow trout, first in experimental groups^11^, but also in farmed populations (unpublished data). These spontaneous masculinisation and their transmission across generations have been characterized in gynogenetic families, where the role of minor genetic factors acting in addition to the major *sdY* sex determination system has been suspected^11,12^. Later, Guyomard et al.^13^ (2014) detected QTL associated to masculinisation in two doubled haploid gynogenetic trout families. In addition to this genetic control, a few studies have also shown that high temperature treatments applied before the sexual differentiation and at the period of thermosensitivity can modulate sex differentiation and enhance the frequency of maleness in rainbow trout^14,15^, as shown in some other species including for instance channel catfish (*Ictalurus punctatus*), Nile tilapia (*Oreochromis niloticus*) and seabass (*Dicentrarchus labrax*)^16,17^. Interestingly, in most of the studies in trout, intermediate phenotypes (intersex individuals with only partial masculinisation of gonads) were recorded.

In rainbow trout farming, the production of all-female populations is advantageous due to their late sexual maturation (most usually at 2 years-old) compared to males (about 1 year of age). Indeed, maturing males exhibit a reduced growth and flesh quality (reduction of muscle lipid content and discoloration) and are more sensitive to fungal saprolegnosis. All-female stocks are produced by mating standard XX-females with sex-reversed XX-males. Sex reversal is obtained by feeding young trout fry with a diet containing a masculinising hormone, the 17α-methyltestosterone before the sexual differentiation period^18^. The administration of hormones to obtain sex-reversed males is carried out under strict veterinary control according to the European directive (99/22/CE, 29 April), and treated animals are euthanized after reproduction and discarded from the food chain.

Therefore, there is a dual interest in investigating factors that govern the spontaneous maleness in trout. Depending on the nature of these factors, they could be exploited either to reduce the occurrence of undesirable males observed in all-females populations or to produce sex-reversed males without using hormones, an asset for more sustainable aquaculture.

To further investigate the genetic architecture of the spontaneous maleness, we performed a Genome-Wide Association Study (GWAS) in a French commercial rainbow trout population, in which spontaneous maleness has been repeatedly reported. Genotype information from a medium-density trout genotyping array and whole genome sequencing (WGS) was combined to accurately map QTL- and identify candidate genes responsible for spontaneous maleness.

## Methods

### Ethics statement

Fish were reared at the farm « Les Fils de Charles Murgat » (Beaufort, France; UE approval number FR 38 032 001) under conditions complying with the Directive 98/58/CE on the protection of animals kept for farming purposes. The fish were euthanized following the approved method used in the farm for animals intended for marketing (electro-narcosis).

Article 3.1 of the EU Directive 2010/63/ EU on the protection of animals used for scientific purposes “*excludes the killing of animals solely for the use of their organs or tissues*” from the procedures covered by the Directive. As such, the experiment was not subject to oversight by an institutional ethic committee.

Tissue sampling and necropsy to observe the gonads were carried out *post-mortem*, with the authorisation of an official veterinarian.

### Fish rearing

In June 2017, gametes were collected from 2.5 years-old breeders that had been previously isolated in light-proofed tanks and exposed to an artificial photoperiod to induce maturation (constant light during two months followed by a 4-months period with partial (8/24 h) lighting, water temperature varied from 11 to 13.5 °C). In total, 50 dams (XX-trout, average body weight of 4.61 ± 0.84 kg) and 50 XX sex-reversed sires (average body weight of 3.28 ± 0.60 kg) were mated according to 5 full-factorial mating designs (10 dams × 10 sires per factorial) to produce all XX-eggs. Fertilized eggs were separated into two batches corresponding to two temperature treatments (25,000 and 45,000 eyed eggs in batch 1 and 2, respectively) and incubated in McDonald-type jars with a bottom-to-top water flow maintained at 10.5 °C until hatching. Hatching rate was 64% and 58% in the two batches, which is in the usual range when using progenitors whose spawning has been shifted by photo-period. At the end of yolk resorption (35 days post-fertilisation, dpf), larvae of batch 1 were kept at 12 °C (control group) while in batch 2, the water temperature was increased by 1 °C/day until it reached 18 °C. The water temperature was then maintained constant for 1,134 degree-days covering the expected window of gonad differentiation^12^. It was then decreased by 1 °C/day during 6 days to the same temperature as in the control group (12 °C) and both groups were then reared between 12 and 14 °C. At 84 dpf, 13,000 fish from each temperature group were randomly sampled and retained for the experiment. In order to prevent too much heterogeneity in size within the groups and any risk of biased sampling at time of phenotyping, each group was temporarily split (at 214 dpf) into two subgroups according to the fish size and kept separated for 37 days, allowing the smaller fish to catch up in size with the bigger fish. Fish were then grouped again until sex was recorded. During the whole experiment, fish were fed a commercial diet according standard recommendations.

### Phenotypic sex recording

In order to determine the phenotypic sex of the XX-offspring, 13,241 fish from control and 18 °C groups (6560 and 6681 fish, respectively) were sexed at 10 month-post fertilisation (mpf) and 6969 more fish were sexed 5 months later (3456 and 3513 fish, respectively). The overall mean body weight of fish was respectively 234 g at 10 mpf and around 860 g at 15 mpf. Fish were euthanized by electro-narcosis, according to standard procedures for commercial trout farming and both gonads were visually examined to determine sex. When necessary, visual observation was completed by observation of the gonads under a binocular magnifier (1079 fish) and, in some cases, by a histological control (23 fish).

Fish were distributed into four sex classes (no matter the degree of maturation) as followed:

- Female, for fish with two ovaries and no visible sign of testis area (illustration in Supplementary Figure S1, panel a).
- Intersex, for fish with either one entirely female gonad and one entirely male gonad, or with both male and female areas in at least one of the gonads (the other gonad may be male, female, intersex or undetermined) (n = 132). The presence of ovarian lamellae was the criterion used to declare a female area in a gonad, whether oocytes were present or not (illustration in Supplementary Figure S1, panel b).
- Male, for fish with two testis, or for five fish, one testis only (the other gonad being undifferentiated) and no visible sign of any ovary area (n = 162) (illustration in Supplementary Figure S1, panel c).

Fish with undifferentiated gonads that could not be sexed after binocular or histological observation were classified as undetermined and removed from the analysis (n = 84).

All intersex individuals (n = 132), all males (n = 162) as well as 858 females with well-developed ovaries were kept for QTL detection (563 from the control group, at both 10 and 15 mpf, and 295 from the 18 °C group at 15 mpf). Sex was recorded as a categorical variable with three levels: sex = 1 for females, sex = 2 for intersex, sex = 3 for males.

### Genotyping and sequencing

Pieces of caudal fin sampled from those 1152 fish were sent to Gentyane genotyping platform (INRAE, Clermont-Ferrand, France) for DNA extraction using the DNAdavance kit from Beckman Coulter following manufacturer instructions. Genotyping was performed with the Axiom Trout Genotyping Array from Thermofisher^19^ that contains 57,501 SNPs.

Quality controls of genotyped SNPs were performed as described in D’Ambrosio et al.^20^, in particular to remove SNPs with probe polymorphism and multiple locations on the genome. In addition, only the 31,811 SNPs with a call rate higher than 0.97, a test of deviation from Hardy–Weinberg equilibrium with a p-value > 0.0001 and a minor allele frequency (MAF) higher than 0.05 were kept for further analyses. Individuals with a call rate lower than 0.90 were removed from the genotype dataset (n = 5). All missing genotypes were imputed using the FImpute software^21^ (version 2.2) in order to get the full 31,811 SNPs genotypes for all the animals considered in the analyses.

Samples of caudal fin were also collected from a set of 60 females (the 50 dams + 10 relatives) for DNA extraction and whole genome sequencing. DNA was extracted with a Promega Relia Prep dDNA tissue miniprep system (A2051) following manufacturer instructions. DNA sequencing was performed at the GeT-PlaGe core facility (INRAE, Toulouse, France, https://get.genotoul.fr/en/). The DNA was prepared according to Illumina protocols using the Illumina TruSeq Nano DNA HT Library Prep Kit. Briefly, DNA was fragmented by sonication, size selection of fragments was performed using SPB beads (kit beads) and adaptors were ligated for latter identification of individuals and fragment were pooled to be sequenced. Library quality was assessed using Advanced Analytical Fragment Analyser and libraries were quantified by QPCR using Kapa Library Quantification Kit. DNA sequencing was performed on an Illumina NovaSeq6000 using a paired-end read length of 2 × 150 bp with the Illumina NovaSeq6000 S4 Reagent Kits.

After sequence mapping on the reference genome assembly^22,23^ (bwa mem v0.7.17, GCA_002163495.1), 34,064,394 variants were obtained using a homemade pipeline (https://forgemia.inra.fr/bios4biol/workflows/tree/master/Snakemake/IMAGE_calling) based on three variant calling tools (GATK v3.7.0, FreeBayes v1.2.0 and SAMtools mpileup v1.8.0). Quality controls and filtering were performed using vcftools^24^ (version 1.15). First, indels and SNPs on un-located contigs or mitochondrial DNA were removed. Only the 21,904,314 bi-allelic SNPs located on identified chromosomes were considered for further analyses. Then, a filtering was performed on variant coverage and variants with either less than 10X reads or more than 50X reads (considered as putative duplicated regions) were removed. Variants with more than 58 individuals being homozygous for either the reference or the alternative allele were removed from the analysis. In other words, 14,478,077 variants with at least two individuals different from the 58 others (either heterozygous or homozygous for the alternative alleles) remained. Finally, only the 8,784,147 variants with a MAF equal or above 10% were kept for imputation and genome wide association studies (GWAS).

### Imputation to whole-genome-sequence

Imputation of the 31K SNPs genotypes of the progeny to whole-genome sequence (WGS) was performed chromosome by chromosome using the latest version of FImpute software^21^ (version 3.0) based on the reference population constituted by the 60 sequenced females. After imputation, 8,765,613 SNPs with a MAF higher than 1% were kept for the WGS analysis. In order to reduce the variant dataset to produce a genomic relationship matrix, SNPs in linkage disequilibrium were filtered out with the indep-pairwise option of the PLINK software^25^ (version 1.09). The filtering was performed first on 50 bp sliding windows. In every window, the r^2^ between each pair of SNPs was calculated and one SNP of the pair was removed if r^2^ was higher than 0.7. A second round of filtering was performed using 100 bp sliding windows and r^2^ higher than 0.3. The reduced dataset obtained was composed of 275,283 SNP.

### Genome wide association studies

#### Models

GWAS were performed at the genotyping level on the 31K SNPs dataset and at the WGS level on the 8.7 million SNPs dataset, using two different approaches, a marker-by-marker analysis and a Bayesian Stochastic Search Variable Selection approach.

The first GWAS analysis was performed with the GCTA software^26^ that performs a marker-by-marker analysis under a mixed linear model with a correction for data structure based on a genomic relationship matrix^27^ (GRM).

The model used in this first GWAS is described by the Eq. (1):

1 $${{y}_{i}}=\mu +{{T}_{i}}+{{a}_{j}}{{x}_{ij}}+{{u}_{i}}+{{\varepsilon }_{i}}$$

with ${y}_{i}$ the observed phenotype for the i^th^ individual (sex in 3 levels), $\mu$ the overall mean in the population, ${T}_{i}$ the fixed effect of the temperature treatment (2 levels), ${a}_{j}$ the additive effect of the reference allele of the candidate SNP (j) to be tested as fixed effect for sex association and ${x}_{ij}$ the reference allele count (0, 1, or 2) for the SNP j for individual i, ${u}_{i}$ is the random polygenic additive value of individual i and ${\varepsilon }_{i}$ the residual effect for individual i. The vector of residual effects is normally and independently distributed ${\varvec{\varepsilon}}\sim N\left(0, {\varvec{I}}{\sigma }_{e}^{2}\right)$ with σ^2^_e_ the residual variance. The random vector of polygenic effects follows a normal distribution ${\varvec{u}} \sim N\left(0, {\varvec{G}}{\sigma }_{g}^{2}\right)$ with ${\sigma }_{g}^{2}$ the estimated genetic variance and **G** a GRM constructed using only SNPs information. We first performed a GWAS on the 31K SNPs level with a GRM constructed using all 31K SNPs included in the model, this analysis will be referred to as GCTA-chip (see Table 1). In order to refine the position and size of the QTL detected by the analysis at the 31K SNPs level, we performed the same GWAS using the WGS dataset. This GWAS at the sequence level was performed on the whole 8.7 million SNPs with a GRM constructed with 275K SNPs according to Yang et al.^27^. This GWAS will be referred to as GCTA-seq (Table 1).

**Table 1:** Summary of GWAS analyses used to detect QTLs associated with spontaneous maleness in XX-rainbow trout.

Name	Software	Number of SNPs in the analysis	Number of SNPs in GRM	π
GCTA_chip	GCTA	30,811	30,811	–
GCTA_seq	GCTA	8,765,613	275,283	–
BCπ-chip	BESSiE	30,811	275,283	1%
BCπ-seq	BESSiE	21,149	275,283	0.02%

π is the proportion of SNPs with a non-zero-effect included at each cycle k of the MCMC algorithm.

*GRM* genomic relationship matrix.

The second GWAS approach was performed using the Bayesian Stochastic Search Variable Selection approach BayesCπ^28^ implemented in the BESSiE software^29^ (version 1.0). In this BayesCπ approach, at each iteration, a proportion π of SNP is assumed to have a non-zero-effect on the trait, thus at each iteration the number of SNP effects to be estimated is lower than the number of phenotypic records. The statistical model used is defined according to Eq. (2):

2 $${y}_{i}=\mu + {T}_{i}+ {u}_{i}+ \sum_{j=1}^{n}{{\delta }_{jk}a}_{j}{g}_{ij}+ {\varepsilon }_{ik}$$

with ${y}_{i}$ the observed phenotype the ith individual (sex in 3 levels), μ the overall mean in the population, ${T}_{i}$ the fixed effect of the temperature treatment (2 levels), ${u}_{i}$, the additive polygenic effect for individual i, ${a}_{j}$ is the additive effect of the reference allele for the jth SNP with its genotype for individual i (${g}_{ij}$) coded as 0, 1 or 2 and n the number of SNPs in the analysis; ${\delta }_{jk}$ is the indicator variable for the non-zero effect of the jth SNP at the kth iteration; ${\varepsilon }_{ik}$ the residual effect for the ith individual at the kth iteration. As in the previous model, the random vector of polygenic effects follows a normal distribution ${\varvec{u}} \sim N\left(0, {\varvec{G}}{\sigma }_{g}^{2}\right)$ with ${\sigma }_{g}^{2}$ the estimated genetic variance and **G** the GRM constructed using information from the 275K SNPs. The vector of residual effects is normally and independently distributed ${\varvec{\varepsilon}}\sim N\left(0, {\varvec{I}}{\sigma }_{e}^{2}\right)$ with σ^2^_e_ the residual variance.

At each cycle k, the decision to include the SNP j in the model depends on the indicator variable δ_jk_, with the effect (${a}_{j}$) of the SNP j estimated if δ_jk_ is equal to 1, and not estimated if δ_jk_ is equal to 0. This indicator variable^28^ is sampled from a binomial distribution with a probability π that δ_jk_ is equal to 1 (the SNP as a non-zero-effect) and a probability 1 − π that δ_jk_ is equal to 0.

As for the marker-by-marker approach, a first GWAS using the BayesCπ model was performed on the 31K SNPs level with parameters determined in order to have 1% of the markers included in the model at each cycle. This analysis will be referred to as BCπ-chip (Table 1). To refine the localisation of the QTL, the second GWAS was performed at the WGS level using only SNPs located on a portion of one chromosome (selected using the results of the GCTA-seq analysis) with the effect of all other SNPs (from this chromosome and the others) included in the polygenic effect calculated with the GRM based on 275K SNPs. For this analysis, which will be referred to as BCπ-seq (Table 1), the proportion π of SNPs to be included at each cycle was constrained to 0.02% (i.e. 3 SNPs included at each iteration).

Both BayesCπ analyses were performed with a MCMC algorithm. In total, 500,000 iterations were used for the BCπ-chip and 1 million iterations were used for the BCπ-seq, with, for both analyses, a burn-in period of 50,000 cycles and results saved every 20 cycles. In order to control the reproducibility of our analyses, both were run twice, with two different seeds for the random number generator to initialize MCMC algorithm. Convergence was assessed by visual inspection of trace plots of the SNP effects and variance components estimates for all different seeds.

#### Estimation of genetic parameters

Variance components and heritability were estimated using the average information of restricted maximum likelihood analysis (AI-REML) implemented in GCTA software^26^ with the model described in Eq. (1). The two GRMs built with 31K and 257K SNPs for GWAS were used to estimate the genetic parameters at the genome and the sequence level, respectively.

The values obtained with the 31K SNPs GRM were used as prior for both BayesCπ models and the genetic variance ${(\sigma }_{a}^{2})$ was calculated as the sum of the polygenic variance (${\sigma }_{u }^{2}$, estimated by BESSiE) and the genomic variance $({\sigma }_{g}^{2})$ estimated by n SNPs calculated according to Eq. (3):

3 $${{\sigma }_{g}^{2}}= \sum_{i=1}^{n}2{p}_{i}{q}_{i}{a}_{i}^{2}$$

with ${p}_{i}$ and ${q}_{i}$ the allele frequencies for the i^th^ SNP and ${a}_{i}$ the estimated additive effect of the i^th^ SNP.

Genomic heritability (h^2^_g_) was estimated as $$h_{g}^{2} = {\raise0.7ex\hbox{${\sigma _{a}^{2} }$} \!\mathord{\left/ {\vphantom {{\sigma _{a}^{2} } {\left( {\sigma _{a}^{2} + \sigma _{e}^{2} } \right)}}}\right.\kern-\nulldelimiterspace} \!\lower0.7ex\hbox{${\left( {\sigma _{a}^{2} + \sigma _{e}^{2} } \right)}$}}$$ with $${\sigma }_{a}^{2}$$ the estimated genetic variance and $${\sigma }_{e}^{2}$$ the residual variance.

#### QTL definition

For the GCTA-chip and GCTA-seq analysis, we determined chromosome-wide suggestive and genome-wide significance thresholds using a Bonferroni correction with α = 1%, i.e. genome-wide threshold = − log_10_(α/n) and chromosome-wide threshold = − log_10_(α/[n/30]), with n the number of SNPs in the analysis (30,811 or 8.7 millions). Only SNPs with − log_10_(P-value) over the chromosome-wide threshold for GCTA-chip and over the genome-wide threshold for GCTA-seq were considered. For each QTL with a peak SNP value over the significance threshold, approximate confidence intervals (CIs) were estimated using a drop-off limit^30^ of 1.5 unit of − log_10_ (P-value) and a maximum distance of 1 Mb for the GCTA-chip and 200 kb for the GCTA-seq between two successive SNPs, with − log_10_(P-value) over the drop-off limit starting from the position of the peak SNP.

In the GCTA-seq, when two successive QTLs were detected at a distance lower than 50 bp and with less than 250 kb between their peak SNPs, the CIs were cumulated in a credibility interval (see Supplementary Table S1 for detailed CIs).

For the Bayesian approaches, the degree of association between a SNP and the phenotype was assessed using the Bayes Factor^31^ (BF):

4 $$BF= \frac{{P}_{j}/(1-{P}_{j})}{\pi /(1-\pi )}$$

with ${P}_{j}$ the probability of the SNP j having a zero effect and π the proportion of SNPs having a non-zero effect on the trait (in our case π = 1% for BCπ-chip and π = 0.02% for BCπ-seq). BF was transformed into logBF computed as twice the natural logarithm in order to obtain values of the same usual range as the P-value, thus facilitating the visual appraisal of QTL and the comparison between methods^32^. In order to define QTL with the BayesCπ approaches, we considered two categories to classify the strength of the evidence in favour of a QTL^33^: strong evidence for 8 ≤ logBF < 10 and very strong evidence for logBF ≥ 10. Because the BF is not a statistic test, true confidence intervals cannot be derived, but credibility intervals can be built as defined in Michenet et al.^34^. Credibility intervals were determined using the threshold logBF ≥ 8 for defining a peak SNP showing evidence for a QTL in either a BCπ-chip or a BCπ-seq analysis. For the BCπ-chip approach, the credibility interval included all SNPs with a logBF > 3 within a 1 Mb sliding window from the peak SNP. For the BCπ-seq approach, it included all SNPs with a logBF > 5 within a 200 kb sliding window from the peak SNP.

The proportion of the total genetic variance explained by each SNP was derived from the Bayesian analyses and calculated according to the ratio $\frac{2{p}_{i}{q}_{i}{a}_{i}^{2}}{{\sigma }_{a}^{2}}.$ The proportions of genetic variance explained by all the SNPs within the credibility interval of a QTL were cumulated to estimate the proportion of variance explained by the QTL.

#### Candidate genes and SNP annotation

Candidate genes located within a reduced interval determined as the intersection of the confidence and the credibility intervals of the main QTLs were listed from the NCBI *Oncorhynchus mykiss* Annotation Release 100 (GCF_002163495.1).

Annotation of SNPs within those intervals was performed using the SNPEff software^35^ (version 4.3) with the NCBI *Oncorhynchus mykiss* Annotation Release 100 (GCA_002163495.1)^22,23^ as a reference. SNPs were then filtered according to their estimated putative impact, all SNPs with a modifier putative impact were filtered out and only SNP with a low, moderate, or high putative impact were conserved.

#### Pedigree and dams’ genotypes

**Figure 1:**
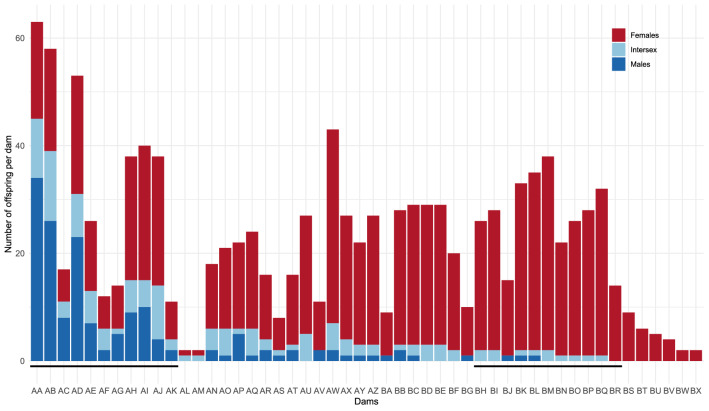
Total number of genotyped offspring (females, males and intersex) for each of the 50 dams (AA to BX) used in the mating design. The 22 dams with more than 10 genotyped offspring and extreme masculinisation rate selected for in-depth analysis of their genotypes are identified by a black underlying line.

Genomic pedigree information was recovered using identity by descent (IBD) estimates from PLINK software^25^ (version 1.9) based on information from the 31K SNPs. The percentage of masculinised fish in the genotyped offspring of each dam was calculated (see Supplementary Table S2). The 50 dams used in the mating scheme were labelled according to the masculinisation rate in their genotyped offspring, with the AA-dam and the BX-dam having the higher and the lower proportions of masculinised offspring, respectively (Fig. 1). Among the 40 dams with at least 10 progeny recorded, 22 dams with extreme proportions of the different phenotypes in their genotyped progeny were selected: the 11 dams (labelled from BH to BR) that had less than 8% of masculinised offspring and the 11 dams (labelled from AA to AK) that had more than 35% of masculinised offspring. Those 22 dams with contrasted masculinisation rates in their offspring were selected for in-depth analysis of their genotypes at the QTL regions.

## Results

### Phenotyping, genotyping and pedigree

In this experiment, we phenotyped 20,210 XX-rainbow trout from the French trout farm « *Les Fils de Charles Murgat* ». Among them 98.1% were females (Table 2) and only 1.4% of fish were partially (intersex) or fully masculinised. From the 294 masculinised individuals, 45.0% were intersex, with both female and male gonadal tissues. Among these intersex individuals, 60.6% had a right gonad completely masculinised or more masculinised than the left gonad. A significantly (p = 3.201e^−12^) higher maleness (males + intersex) was observed in the group that was reared at 12° (2.0%) than in the group reared at 18° (0.9%) (Table 2).

**Table 2:** Phenotyping and genotyping dataset from the XX-rainbow trout produced at “*Les fils de Charles Murgat”* farm.

Temperature treatment (°C) and age at phenotyping	12° 10 mpf	12° 15 mpf	Total 12°	18° 10 mpf	18° 15 mpf	Total 18°	Total
Total number of phenotyped fish	**6560**	**3456**	**10,016**	**6681**	**3513**	**10,194**	**20,210**
Total number of genotyped fish	**415**	**353**	**768**	**41**	**343**	**384**	**1152**
Phenotyped and genotyped females	6435 (305)	3336 (258)	**9771** **(563)**	6616 (0)	3445 (295)	**10,061** **(295)**	**19,832** **(858)**
Phenotyped and genotyped intersex	45	33	**78**	24	30	**54**	**132**
Phenotyped and genotyped males	65	62	**127**	17	18	**35**	**162**
Phenotyped undetermined individuals	15	25	**40**	24	20	**44**	**84**
Proportion of masculinised individuals (intersex + males)	1.68%	2.75%	**2.04%**	0.61%	1.37%	**0.87%**	**1.45%**

*mpf* months post-fertilisation.

Total number of fish are highlighted in bold.

From the 1152 genotyped XX-rainbow trout, 1139 fish genotyped for 30,811 SNPs were retained after quality controls. Pedigree was recovered for all of them but four. In average, the 50 dams had 22.7 genotyped offspring (min = 2, max = 63). The average percentage of masculinised fish in the genotyped offspring sample was 20.7%, ranging from 0% for eight dams to 71.4% for the AA-dam (see Fig. 1 and Supplementary Table S2). The 11 dams (labelled from dam AA to dam AK, Fig. 1) that had the highest proportion of masculinised offspring in the genotyped sample bred 370 fish and accounted for 68% of masculinised genotyped offspring. Conversely, the 11 dams with a low masculinised rate among their genotyped offspring sample (dam BH to dam BR, Fig. 1) accounted for only 5% of the masculinised genotyped offspring. Among those 22 dams, we detected two couples of related (IBD coefficients of 0.36) dams (AA-BK and AI-BR). In those two pairs, one “sister” produced a highly masculinised offspring (71.4% for AA or 37.5% for AI) while the other “sister” produced only a few masculinised individuals (6.1% for BK or 0% for BR).

### Estimation of genetic parameters for spontaneous maleness in XX-rainbow trout

Table 3 presents the variance components and the heritability of the sexual phenotype estimated with the different genomic models using either the 30,811 SNPs of the chip or the 8,784,147 SNPs from the WGS. The estimated heritability of maleness was high, ranging from 0.48 with the GCTA-chip analysis to 0.62 with the GCTA-seq analysis. The estimated genetic variance (${\sigma }_{a}^{2}$) was consistent across all models, with the lower value obtained for GCTA-chip (0.21), intermediate values obtained with BayesCπ models (0.24 and 0.25 for BCπ-chip and BCπ-seq, respectively) and the highest value (0.26) obtained with the GCTA-seq analysis. The estimate values of genetic variance increased with the number of SNPs (from 31K to WGS) included in the model and with the addition of a polygenic effect under a Bayesian approach, showing that the 31K SNPs genotyped were insufficient to capture all the genetic variation. Therefore, the actual heritability of maleness in rainbow trout is expected to be about 0.6.

**Table 3:** Estimates of genetic and genomic parameters for spontaneous maleness under the different statistical models.

Analysis	${h}_{g}^{2}$± SE	${\sigma }_{p}^{2}$± SE	${\sigma }_{e}^{2}$± SE	${\sigma }_{a}^{2}$± SE	${\sigma }_{u}^{2}$± SE	${\sigma }_{g }^{2}$± SE
GCTA-chip	0.48 ± 0.04	0.43 ± 0.02	0.22 ± 0.01	0.21 ± 0.03	-	-
BCπ-chip*	0.56 ± 0.05	0.43 ± 0.02	0.19 ± 0.02	0.24 ± 0.03	0.10 ± 0.03	0.14 ± 0.03
GCTA-seq	0.62 ± 0.06	0.42 ± 0.02	0.16 ± 0.02	0.26 ± 0.03	-	-
BCπ-seq*	0.59 ± 0.05	0.44 ± 0.02	0.17 ± 0.02	0.25 ± 0.03	0.21 ± 0.03	0.04 ± 0.01

h^2^_G_: genomic heritability, calculated as ${\sigma }_{a }^{2}/ ({\sigma }_{a}^{2}+ {\sigma }_{e}^{2})$. ${\sigma }_{a}^{2}$: total genetic variance ${(= \sigma }_{u}^{2}+ {\sigma }_{g}^{2}$), ${\sigma }_{u}^{2}$: polygenic variance, ${\sigma }_{g}^{2}$: genetic variance explained by SNPs, ${\sigma }_{e}^{2}$: residual variance, ${\sigma }_{p}^{2}$: phenotypic variance ${(=\sigma }_{a}^{2}+ {\sigma }_{e}^{2}$).

*Value of one MCMC chain among the two used for GWAS, the chain with the closest final π to the 1% or 0.02% was chosen.

Based on the BCπ-chip model, we estimated that the genetic variance (${\sigma }_{g}^{2}$) explained by the 31K SNPs spanning the whole genome accounted for only 58% of the total genetic variance of sex (${\sigma }_{a}^{2}$, Table 3). Using the BCπ-seq model allowed to focus the analysis on the most relevant genomic regions and showed that the genetic variance (${\sigma }_{g}^{2}$) explained by the sequence segment spanning the 4 Mb located between 62 and 66 Mb on Omy1 accounted for 16% of the total genetic variance of sex (${\sigma }_{a}^{2}$, Table 3).

### Genome wide association studies for maleness in XX-female rainbow trout

#### GCTA-chip and BCπ-chip approaches at the 31K genotyping level

Based on the 31K SNPs, we detected four QTLs associated with maleness on three different chromosomes (Omy1, 12 and 20) with the GCTA and the BayesCπ analyses (Table 4). With the GCTA-chip analysis, we detected two QTL on Omy1, the first one being suggestive only and explaining less than 0.2% of the total genetic variance. The second QTL was significant at the genome-wide level, under GCTA-chip analysis (− log_10 _(P-value) = 11.1) and had a very strong evidence under BCπ-chip analysis (logBF = 11.3) (Table 4). Even if the two peak SNPs of this QTL differed across the two GWAS approaches, the GCTA peak SNP being located 212 kb before the BCπ peak SNP, they were in close vicinity with only three markers in-between the two peak SNPs. This second QTL explained 3.9% of the total genetic variance. We did not distinguish the two QTL with the BCπ-chip approach, as the credibility interval estimated with BCπ-chip was 1.7 Mb and contained the two peak SNPs detected with GCTA-chip.

**Table 4:** Detection of QTLs associated with spontaneous maleness in XX-rainbow trout with GCTA-chip and BCπ-chip methods.

Chromosome	QTL start (Mb)	QTL end (Mb)	Peak SNP name	Peak SNP position (Mb)	Peak − log_10_(P) or logBF	%${\sigma }_{a}^{2}$, explained by QTL	Method
1	62.93206	63.73809	Affx-88953453	63.53900	7.9	–	GCTA-chip
1	64.42011	64.63252	Affx-88916383	64.42011	11.1	–	GCTA-chip
1	63.45244	65.16399	Affx-88950822	64.63252	11.3	3.86%	BCπ-chip
12	6.10355	6.79990	Affx-88940013	6.79990	8.1	0.62%	BCπ-chip
20	31.35239	31.35239	Affx-88916019	31.35239	8.2	0.40%	BCπ-chip

The QTL start and end positions correspond to the confidence and credibility intervals for GCTA-chip and BCπ-chip respectively. For BCπ-chip, the logBF is calculated as twice the natural logarithm of Bayes Factor. % ${\sigma }_{a}^{2}$: proportion of total genetic variance explained by the QTL calculated as the sum of the variance estimated by the BCπ-chip analysis of all SNPs within the QTL credibility interval.

The two other QTLs located on Omy12 and Omy20 were detected using the BCπ-chip approach only (Table 4). The QTL located on Omy12 explained 0.6% of the total genetic variance. The QTL on Omy20, defined by a single SNP located at 31.352 Mb, explained 0.4% of the total genetic variance. This QTL was suggestive at 5% at the chromosome-wide level under GCTA-chip analysis (-log_10_(P-value) = 4.2).

#### GCTA-seq and BCπ-seq approaches at the whole genome sequence level

Using the WGS data and GCTA-seq approach, significant QTLs were detected only on Omy1, in a restricted region (in-between 62 and 66 Mb). In total six peak SNPs corresponding to six putative QTLs (see Supplementary Table S1 and Supplementary Figure S2) were detected in this region. Therefore, the BCπ-seq approach was performed considering only the 21 K SNP spanning this 4 Mb-window on Omy1 in addition to a genome-wide polygenic component. Using this approach, we identified only two QTLs (Table 5 and Supplementary Figure S3). Out of the six putative QTLs detected by the GCTA-seq analysis, the first one was not confirmed with the BCπ-seq analyses. Therefore, it will not be further discussed, as it is likely that the association is only due to linkage disequilibrium with the SNPs included in the second QTL region. The second putative QTL detected between 63.229 and 63.774 Mb with the GCTA-seq analysis was also detected with the BCπ-seq analyses in a reduced credibility interval (between 63.459 and 63.556 Mb, Table 5); it was hereafter considered as the first true QTL on Omy1.

**Table 5:** Summary of the characteristics of the two QTLs associated with maleness in XX-rainbow trout detected using GCTA-seq and BCπ-seq methods.

Chromosome	QTL start (Mb)	QTL end (Mb)	QTL size (kb)	Peak SNP name	Peak SNP position (Mb)	Peak SNP − log_10_(P) or logBF	% ${\sigma }_{a}^{2}$ explained by QTL	Method
1	63.229379	63.774478	545.1	Omy1-63493395	63.493395	10.61	–	GCTA-seq
1	64.360291	64.707100	341.6	Omy1-64597800	64.597800	13.74	–	GCTA-seq
1	63.459553	63.555809	96.27	Omy1-63520900	63.542090	12.97	0.56%	BCπ-seq1
1	64.266321	64.649580	383.26	Omy1-64607018	64.607018	14.67	13.55%	BCπ-seq1
1	63.459545	63.727071	267.53	Omy1-63520900	63.542090	11.55	0.51%	BCπ-seq2
1	64.087947	64.632497	544.55	Omy1-64610233	64.610233	17.32	14.56%	BCπ-seq2

For GCTA-seq, the start and stop positions of the first QTL correspond to a CI estimated with a drop-off method and the start and stop positions of the second QTL correspond to the credibility interval estimated as the union of confidence intervals of four QTLs. The BCπ-seq1 and BCπ-seq2 correspond to the same analysis run with two different seeds for MCMC initialization. Significance of peak SNP is calculated as the –log_10_(P-value) for GCTA-seq, and as logBF estimated as twice the natural logarithm of Bayes Factor for BCπ-seq. % ${\sigma }_{a}^{2}$: proportion of total genetic variance explained by the QTL calculated as the sum of the variance estimated by BCπ-seq analyses of all SNPs within the QTL credibility interval.

The four remaining putative QTLs from the GCTA-seq analysis were very close to each other, with less than 250 kb between two successive peak SNPs, and the limits of their confidence intervals (CIs) approximated by a drop-off approach were all distant from less than 3 kb. Therefore these four CIs were fused into a single credibility interval of 342 kb. This second QTL was also detected with the BCπ-seq analyses (Table 5). While the peak SNP of the first QTL was the same under the two BCπ-seq analyses, this was not the case for this second QTL. However, the two peak SNPs were separated by 3.2 kb only and their credibility intervals were strongly overlapping (Table 5).

Based on the average of the two BCπ-seq analyses, the first QTL explained about 0.5% and the second QTL about 14% of the total genetic variance.

At the end of the second QTL, we identified a haplotype block of 15 consecutive SNPs (spanning 745 kb from 64.632011 to 64.632756 Mb) that have a significant effect on masculinisation (all the 15 SNPs have − log_10_(P-value) between 12.2 and 13.6 and among them five SNPs with logBF > 9). Interestingly, for this 15 SNPs-haplotype, eight of the 11 dams with almost no masculinised offspring carried two copies of the reference genome haplotype (the three remaining dams being heterozygous at the15 SNPs). Among the 11 dams with a high rate of masculinised offspring, only three were homozygous for the reference haplotype, six were heterozygous and the last two (labelled AA and AJ in Fig. 1) were homozygous for the alternative haplotype. In the offspring, the homozygous genotype for the alternative haplotype was overrepresented in masculinised fish (51.5% of masculinised fish) and underrepresented in females (8.7% of females). Conversely, the reference haplotype was observed either at the heterozygous or homozygous state for respectively 50.6% and 40.2% of the female progeny.

### Positional candidate genes and SNP annotation

**Figure 2:**
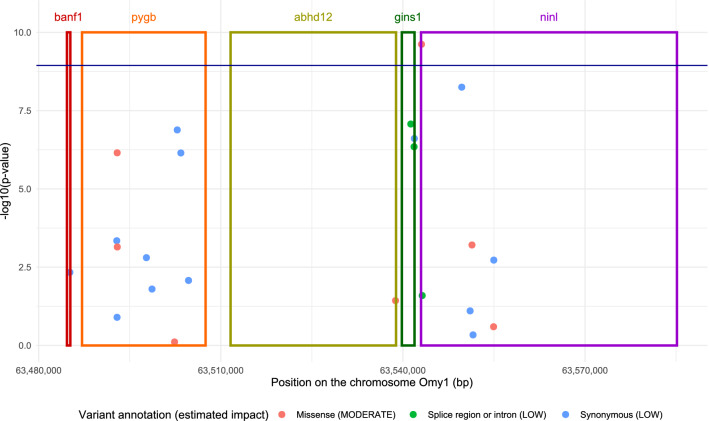
Annotated SNPs located within the first QTL region from 63.549 to 63.556 Mb on Omy1. The significance of SNP effect (dots) is given by the value of – log_10_(p-value) estimated with the GCTA-seq method. The dark blue line corresponds to the 1% threshold at the genome wide level. Only SNPs with at least low putative effects on genes (estimated by SNPEff) are represented. The positions of the five genes located within the QTL region are figured by rectangles: *banf1* (barrier-to-autointegration factor-like), *pygb* (glycogen phosphorylase, brain form), *abhd12* (alpha/beta-Hydrolase domain containing 12), *gins1* (DNA replication complex GINS protein PSF1), *ninl* (ninein-like protein).

Five genes were located within the first QTL region spanning from 63.459 to 63.556 Mb (Supplementary Table S3). The peak SNP from GCTA-seq (at 63.493 Mb) was located within the *pygb* gene (glycogen phosphorylase, brain form, from 63.487 to 63.508 Mb), and the peak SNP from both BCπ-seq runs (6.542 Mb) was located within the *ninl* gene (ninein-like protein, from 63.542 to 63.594 Mb). Among the 669 annotated SNPs spanning the QTL region, only 23 SNPs were indicated with either a low (16 SNPs) or a moderate (7 SNPs) potential effect on gene expressions when annotated with the SNPEff software (See Fig. 2 and Supplementary Table S4). The only SNP that was both significant (− log_10_(P-value) = 9.6) and annotated with a potential moderate effect was located at 63 543 061 bp within the *ninl* gene and annotated as a missense variant (Fig. 2). This SNP was very close to the peak SNP detected by the two BCπ-seq analyses (Table 5).

**Figure 3:**
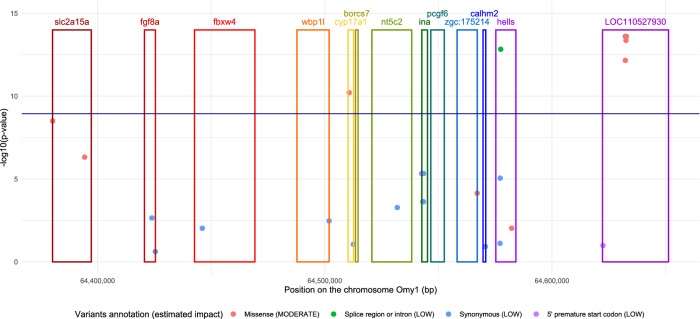
Annotated SNPs located within the second QTL region, from 64.360 to 64.633 Mb on Omy1. The significance of SNP effect (dots) is given by the – log_10_(p-value) estimated with the GCTA-seq method. The dark blue line corresponds to the 1% threshold at the genome wide level. Only SNPs with at least low putative effects on genes (estimated by SNPEff) are represented. Genes located within this QTL are represented by rectangle: *slc2a15a* (solute carrier family 2, facilitated glucose transporter member 9-like), *fgf8* (fibroblast growth factor 8), *fbxw4* (F-box/WD repeat-containing protein 4), *wbp1l* (WW domain binding protein 1-like), *cyp17a1* (Cytochrome P450 Family 17 Subfamily A Member 1), *borcs7* (BLOC-1-reltaed complex subunit 7), *nt5c2* (cytosolic purine 5′-nucleotidase), *ina* (alpha-internexin), *pcgf6* (polycomb group RING finger protein 6), *zgc:175214* (RING finger protein 122-like), *calhm2* (calcium homeostasis modulator protein 2), *hells* (lymphocyte-specific helicase-like), LOC110527930 (uncharacterized protein).

Within the second QTL region spanning from 64.360 to 64.633 Mb, 13 genes were detected (Supplementary Table S3). The three peak SNPs (Table 5) identified by the different WGS analyses were all located in the intergenic region between the *hells* gene (helicase, lymphoid specific) and an uncharacterized protein (LOC110527930, see Supplementary Table S3). Among the 1498 SNPs annotated in the QTL region, only 44 had a potential effect (moderate for 15 SNPs and low for 29 SNPs) on gene expression (Fig. 3 and Supplementary table S5). Among those 44 SNPs, 12 with a potential effect on the 3 genes *cyp17a1* (Cytochrome P450 Family 17 Subfamily A Member 1), *hells*, and LOC110527930 were also significant (− log_10_(P-value) > 10) based on GCTA-seq analysis (Fig. 3). One of those 12 significant SNPs (− log_10_(P-value) = 10.2) was annotated as a missense variant within the *cyp17a1* gene (Fig. 3). Two others SNPs (− log_10_(P-value) = 12.8 and 11.7) were annotated for a low potential effect on the *hells* gene expression as they corresponded respectively to a variant within a splice region or an intron of the gene for the first SNP and to a synonymous mutation for the second SNP (see Supplementary Table S5). Finally, the remaining nine SNPs annotated with either low (2 SNPs) or moderate (7 SNPs) potential effects on the expression of the LOC110527930 uncharacterized protein (see Supplementary Table S5) were SNPs from the 15 SNPs-haplotype block previously identified. All the seven SNPs annotated for moderate effects were missense variants. Among them, two SNPs, located at 64,632,546 bp and at 64,632,583 bp (Fig. 3), seemed to be good candidates for the causative mutation as they were both significant in the GCTA-seq and one BCπ-seq analysis (− log_10_(P-value) = 13.6 and logBF > 11).

## Discussion

To the best of our knowledge, this study is the first one to investigate the determinism of spontaneous masculinisation in XX rainbow trout through GWAS. The analysis was carried out in a rainbow trout commercial line reared either under standard temperatures (12–14.5 °C) or after a high temperature treatment during early stages.

The overall masculinisation rate was 1.45%, which is in the range of values reported by several French farmers (unpublished data). Unexpectedly, the masculinisation rate was significantly higher in the group reared at the 12 °C than for the group exposed to the high temperature treatment (18 °C). This result is in contradiction with the results reported by Valdivia et al*.*^15^ (2014) who observed a masculinising effect of early hot temperature treatment (18 °C vs 12 °C). However, in their experiment, the temperature treatment was applied a little bit earlier during development (at 32 dpf, instead of 35 dpf), so the temperature treatment applied in our study might have not completely overlap the critical thermosensitive period of sex differenciation^36,37^, inducing a repression instead of an induction of masculinisation. This hypothesis deserves further investigation to be confirmed. Different genetic backgrounds could also contribute to the observed differences in response to thermal treatment, as the families tested in Validivia et al.^15^ (2014) belonged to the INRA XX-*mal* lineage, an experimental line different from the commercial population used in this study. The hypothesis is further supported by the fact that distinct maleness QTLs were detected in INRA XX-*mal* families^13^ and in this study (see below), suggesting that masculinising factors may differ according to populations, resulting in differences in susceptibility to temperature. Indeed, a range of sex ratios in response to temperature treatments have been reported in rainbow trout populations with various genetic backgrounds^14,38^. In other fish species such as Atlantic silverside^39^ or Nile tilapia^40^, sex differentiation is also known to depend both on the water temperature and on the genetic background of the population.

Among masculinised individuals, the extent of the gonad masculinisation was variable, with about 40 and 61% of intersex fish in the 12 °C and 18 °C groups, respectively. Within those intersex individuals, whatever the rearing temperature at early stages, the right gonad was very often more masculinised than the left gonad. This particularity was first described by Quillet et al. (2004)^41^ and confirmed by Valdivia et al. (2013, 2014)^12,15^, in families originating from the INRA XX-*mal* carrying line. Under standard rearing conditions, the left–right asymmetry (LR) is almost undetectable, as intersex fish remain rare and poorly described. However, the observation of such an asymmetry in another rainbow trout population suggests that this a general feature in rainbow trout and that gonads develop in an asymmetrical LR manner in this species, as it has been reported in mammals, birds, amphibians and reptiles^42^ and in some fish^43–45^.

In this study, we reported high genomic heritability estimates for spontaneous maleness (from 0.48 up to 0.62). The QTL we detected explained only a small proportion (up to 14%) of the estimated genetic variance. Using the BCπ-chip method, we estimated that the pan-genomic 31 K SNPs explained 58% of the genetic variance of maleness. The remaining 42% of the genetic variance were probably not captured by the SNPs due to their heterogeneous density distribution across the genome as well as to lack of accuracy their effects (regressed towards 0) with only 1139 phenotyped and genotyped individuals.

Concerning the identification of the genetic determinants of maleness, we detected four QTLs on three different chromosomes, two QTLs on Omy1, one on Omy12 and the last one on Omy20. Both QTLs on Omy12 and Omy20 were only detected in the 31K SNPs analyses and were not confirmed with WGS analyses. The QTL on Omy20 might be the result of a spurious association as it was defined by a single SNP in both BCπ-chip and GCTA-chip analyses. It is worth noting that in their study in gynogenetic families from INRA XX-*mal* line, Guyomard et al.^13^ (2014) detected four QTLs associated with spontaneous maleness on four different chromosomes and that one of them was located on Omy20 (linkage group RT17) too. However, the QTL had a wide confidence interval, covering the entire chromosome, so it is not possible to say if the QTL we detected in the present study on Omy20 is the same. As we did not detect QTL on the three other chromosomes reported in Guyomard et al.^13^ (2014), maleness in rainbow trout with different genetic backgrounds is likely to be controlled by a diversity of underlying genetic mechanisms.

The two main QTLs associated with masculinisation in our population were both detected on Omy1, which has never been reported as associated with neither sex determinism nor maleness in rainbow trout. Using WGS information, we were able to reduce the confidence interval of the first QTL from 806 kb (GCTA-chip, Table 4) down to 545 kb (GCTA-seq analysis) and even 96 kb for the credibility interval estimated with one of the BCπ-seq runs (Table 5). While highly significant, this QTL explained only 0.5% of the total genetic variance of the trait. This low proportion of variance explained may be the result of a strong linkage disequilibrium of those SNPs with some SNPs of the second QTL, which was mapped at a close distance (< 1.2 Mb between the peak SNPs of the two QTLs), making the first QTL an artefact. Within this first QTL region, no gene seems to have a functional role in gonad development; however, this QTL may contain regulating factors involved in the control of genes contained in the second QTL.

For the second QTL, the credibility interval ranged from 268 to 545 kb depending on the WGS analyses (Table 5 and Supplementary Table S1). Based on the BCπ-seq analysis, we estimated that this second QTL explained about 14% of the total genetic variance while its contribution was reduced to less than 4% with the BCπ-chip analysis. It is difficult to know whether the proportion of variance explained was overestimated under the BCπ-seq model or underestimated under the BCπ-chip model because we ignored the covariance between close SNPs estimates when deriving this proportion. Nevertheless, this QTL explains a sufficient part of the genetic variance to deserve specific attention in breeding programs.

Within this main QTL region, three candidate genes possibly involved in spontaneous masculinisation were identified. First, the *fgf8a* gene might be involved in the left–right (LR) asymmetrical gonad development observed in the intersex fish. This gene is part of the fibroblast growth factor family which plays a major role in vertebrate development and has been reported to be fundamental for proper LR asymmetric development of multiples organs (brain, heart and gut) in zebrafish^46^. The second and biologically more convincing candidate gene is *cyp17a1* as it is a major enzyme of the steroidogenic pathway and it is involved in the synthesis of biologically active gonadal androgens and oestrogens. Indeed, *cyp17a1* has been identified as potentially involved in spontaneous maleness in common carp (*Cyprinus carpio*)^47^ and was recently characterized as being involved in the gonad differentiation in zebrafish with complete masculinisation phenotype in *cyp17a1* deficient zebrafish^48^. However, in our analysis, the only SNP with a putative missense effect for this gene was significant in the GCTA-seq analysis only (− log_10_(P-value) = 10.2) and had very low logBF values in both BCπ-seq approach (0 or 2.4). However, even if the *cyp17a1* gene was not within the most significant region of the QTL (located in-between the *hells* gene and LOC110527930) in terms of statistical significance/evidence tests, we cannot not exclude its implication, for instance through long distance regulation of its expression. Based on the GWAS results as well as the SNP annotation, the uncharacterized LOC110527930 protein was the most relevant candidate found in our analysis. Within this gene, we identified a haplotype block of 15 consecutive SNPs (from Omy1-64632011 to Omy1-64632756) that were all significant with the GCTA-seq analysis (− log_10_(P-value) > 12) and presented alternative genotypes for four dams with opposite proportions of masculinised progeny (2 dams with highly masculinised offspring and 2 dams with low masculinised offspring). In addition, seven of those 15 SNPs were annotated with a putative missense effect on the LOC110527930 protein. However, there is no published evidence that this uncharacterized protein could play a role in gonadal sex differentiation and more work is still needed to characterise the protein and its potential role in gonadal differentiation.

The high heritability (up to 0.62) of spontaneous maleness estimated in this study opens up opportunities to manage maleness in all-female trout populations. If the two highly significant QTLs detected on Omy1 in the present population were confirmed to play a role in spontaneous maleness in other rainbow trout populations with diverse genetic origins, the identified SNPs could then be used in a cost-efficient genotyping test to identify female broodstock with a higher propensity to transmit male or intersex phenotype in their progeny and help breeders willing to limit the occurrence of undesirable masculinised individuals in their commercial all-female stocks to discard those breeders from reproduction. In order to optimize the efficiency of such a test, i.e. maximising the detection of masculinised fish while keeping low the number *of true* females, we tested various combinations of SNPs located within the two QTLs identified on Omy1. Within the main QTL (from 64.360 to 64.707 Mb), all significant SNPs would give similar yields, any SNP homozygous for the alternative allele would allow the detection of 44.7 to 51.5% of masculinised fish in our sample, whereas only 6.8 to 13.7% of the homozygous fish for the alternative allele would be females. In particular, using the alternative allele of any SNP from the haplotype block of 15 SNPs described previously (between 64,632,011 and 64,632,756 bp) would allow detecting 51.5% of masculinised fish in our sample. Among those 15 SNPs, the SNP Affx-88950822 is present on the commercial 57K chip and could be of immediate use, provided its effect is confirmed in other trout populations. Every combination of two or more SNPs within this main QTL would yield in a smaller proportion of females discarded but also in a slightly smaller proportion of males identified. Always considering our sample, pairing the SNP Affx-88950822 (or any other SNP from the associated 15-SNPs haploblock) with SNPs from the first QTL on Omy1 (from 63,459 to 63,556 Mb) would slightly increase the test sensitivity (identifying more masculinised fish) without eliminating too many true females. Before applying the same test in other populations, the effect of those SNPs should be confirmed in a large set of populations with diverse genetic backgrounds, or at least, in the target population. Our preliminary results regarding maleness QTLs in either the XX-*mal* families from the INRA experimental population and the population used in this study suggest that this might not be the case.

From a breeder perspective, additional research is needed to improve knowledge about the genetic basis and the environmental factors determining spontaneous maleness in all-female stocks before any industrial application. In addition to the confirmation that QTLs are present in other rainbow trout populations, the expected genetic and phenotypic responses under different thermal regimes and either pedigree-based or marker-assisted selection should be quantified to assess the efficiency of a strategy aiming at limiting the rate of spontaneous males in all-female stocks. Further investigation is also needed with regard to the production of XX sex-reversed male breeders based on the use of spontaneous sexual inversion to prevent the use of hormones. Indeed, the high heritability of maleness suggests that the use of spontaneously masculinised individuals as progenitors of all-female populations would increase the frequency of undesirable masculinised progeny. Combining masculinising genetic factors together with an environmental (temperature) control of gonad masculinisation according to the destination of the fish (broodstock vs all-female production stock) and/or the rearing environment might offer a solution to manage the trade-off. However, because of the overall low masculinisation rates recorded in this study, we were not able to detect any potential interaction between rearing temperature and genotype. We have no suspicion of the existence of such an interaction, as a GWAS with GCTA-chip, carried out with only the individuals from the 12 °C group, detected the same QTL as for the overall population (results not shown). In the current state of knowledge on the effect of temperature on sexual differentiation, it is too early to propose an efficient management of the rearing environment to either enhance or limit spontaneous maleness in rainbow trout. Although the prospect of using hormone-free XX-neomales is very attractive, a potential important drawback may be to fall back into the flesh quality and animal welfare issues (flesh loss of lipids, color and firmness due to precocious maturation; mortality due to *Sparolegnia* fungus) encountered in commercial stocks with XY-males.

In conclusion, in this study we detected minor genetic factors involved in maleness in rainbow trout in the absence of the master gene *sdY*. Two QTLs detected on Omy1 explained up to 15% of the total genetic variance of maleness in the population used in this study and we identified three candidate genes that might be involved in the masculinisation of XX-rainbow trout. Among those three genes, one uncharacterised protein (LOC110527930) was the most relevant candidate and more work would be needed to characterise the potential role of this protein on gonadal differentiation.

## Supplementary information

Supplementary Legends.

Supplementary Information 1.

Supplementary Information 2.

## Data Availability

The datasets for this manuscript are not publicly available because data belongs partly to a private company. The data can be made available for reproduction of the results from Florence Phocas and Charles Murgat Pisciculture on request via a material transfer agreement.
