# Supplementary material for: Genetic determinism of spontaneous masculinisation in XX female rainbow trout: new insights using medium throughput genotyping and whole-genome sequencing
Source: Sci Rep. 2020 Oct 19;10:17693. doi: 10.1038/s41598-020-74757-8 (PMC7573577; doi:10.1038/s41598-020-74757-8)
Supplement: Supplementary file 1 — Supplementary Legends. [file 41598_2020_74757_MOESM1_ESM.docx]

**Genetic determinism of spontaneous masculinisation in XX female rainbow trout: new insights using medium throughput genotyping and whole-genome sequencing**

Clémence Fraslin^1^, Florence Phocas^1^, Anastasia Bestin^2^, Mathieu Charles^1,3^, Maria Bernard^1,3^, Francine Krieg^1^, Nicolas Dechamp^1^, Céline Ciobotaru^1^, Chris Hozé^1,4^, Florent Petitprez^5^, Marine Milhes^6^, Jérôme Lluch^6^, Olivier Bouchez^6^, Charles Poncet^7^, Philippe Hocdé^8^, Pierrick Haffray^2^, Yann Guiguen^9^, and Edwige Quillet^1^

^1^Université Paris-Saclay, INRAE, AgroParisTech, GABI, 78350, Jouy-en-Josas, France

^2^SYSAAF, Station LPGP/INRAE, Campus de Beaulieu, 350002 Rennes, France

^3^INRAE, SIGENAE, 78350 Jouy-en-Josas, France

^4^Allice, MNE 149 rue de Bercy, 75595, Paris, France

^5^Programme Cartes d’Identité des Tumeurs, Ligue Nationale Contre le Cancer, 75013 Paris, France

^6^INRAE, US 1426, GeT-PlaGe, 31326, Castenet-Tolosan, France

^7^INRAE, UMR1095, Gentyane, 63000 Clermont-Ferrand, France

^8^Charles MURGAT Pisciculture, 38270 Beaufort, France

^9^INRAE, LPGP, 35000 Rennes, France

**Supplementary information**

**Supplementary Table S1.** GCTA-seq QTLs definition with their confidence intervals in kb.

**Supplementary Table S2.** Descriptive statistics of the 50 dams’ offspring with the proportion of masculinised offspring and detailed number of offspring according to their phenotypic sex.

**Supplementary Table S3.** Candidate genes. QTL start and end correspond to a reduced interval determined as the intersection of confidence and credibility intervals estimated using both GCTA-seq and BCπ-seq analyses.

**Supplementary Table** S4. Annotated SNPs within the first QTL region. Only SNPs with a moderate or low annotated putative impact (estimated with the SNPEff software) are presented.

**Supplementary Table S5.** Annotated SNPs within the second QTL region. Only SNPs with a moderate or low annotated putative impact (estimated with the SNPEff software) are presented.
