# Supplementary material for: Genetic determinism of spontaneous masculinisation in XX female rainbow trout: new insights using medium throughput genotyping and whole-genome sequencing
Source: Sci Rep. 2020 Oct 19;10:17693. doi: 10.1038/s41598-020-74757-8 (PMC7573577; doi:10.1038/s41598-020-74757-8)
Supplement: Supplementary file 2 — Supplementary Information 1. [file 41598_2020_74757_MOESM2_ESM.pdf]

Genetic determinism of spontaneous masculinisation in XX female rainbow trout: new insights using medium throughput genotyping and whole-genome sequencing

Clémence Fraslin[1$*], Florence Phocas[1*], Anastasia Bestin[2], Mathieu Charles[1,3], Maria Bernard[1,3], Francine Krieg[1], Nicolas Dechamp[1], Céline Ciobotaru[1], Chris Hozé[1,4], Florent Petitprez[5], Marine Milhes[6], Jérôme Lluch[6], Olivier Bouchez[6], Charles Poncet[7], Philippe Hocdé[8], Pierrick Haffray[2], Yann Guiguen[9], and Edwige Quillet[1]

[1]Université Paris-Saclay, INRAE, AgroParisTech, GABI, 78350, Jouy-en-Josas, France
[2]SYSAAF, Station LPGP/INRAE, Campus de Beaulieu, 350002 Rennes, France
[3]INRAE, SIGENAE, 78350 Jouy-en-Josas, France
[4]Allice, MNE 149 rue de Bercy, 75595, Paris, France
[5]Programme Cartes d'Identité des Tumeurs, Ligue Nationale Contre le Cancer, 75013 Paris, France
[6]INRAE, US 1426, GeT-PlaGe, 31326, Castenet-Tolosan, France
[7]INRAE, UMR1095, Gentyane, 63000 Clermont-Ferrand, France
[8]Charles MURGAT Pisciculture, 38270 Beaufort, France
[9]INRAE, LPGP, 35000 Rennes, France

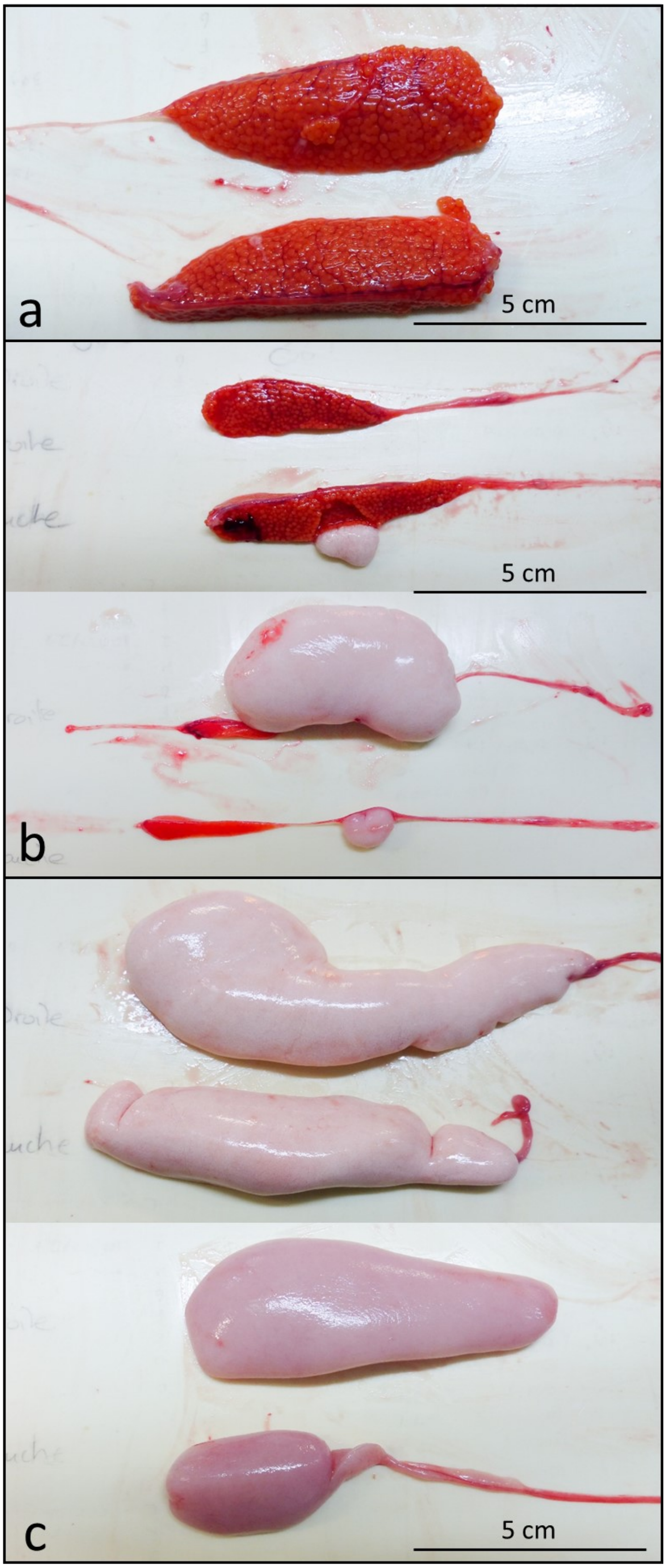

Supplementary Figure S1. Photos of a) female, b) intersex and c) male gonads from phenotyped XX-female rainbow trout from "*Les Fils de Charles Murgat*" French trout farm.

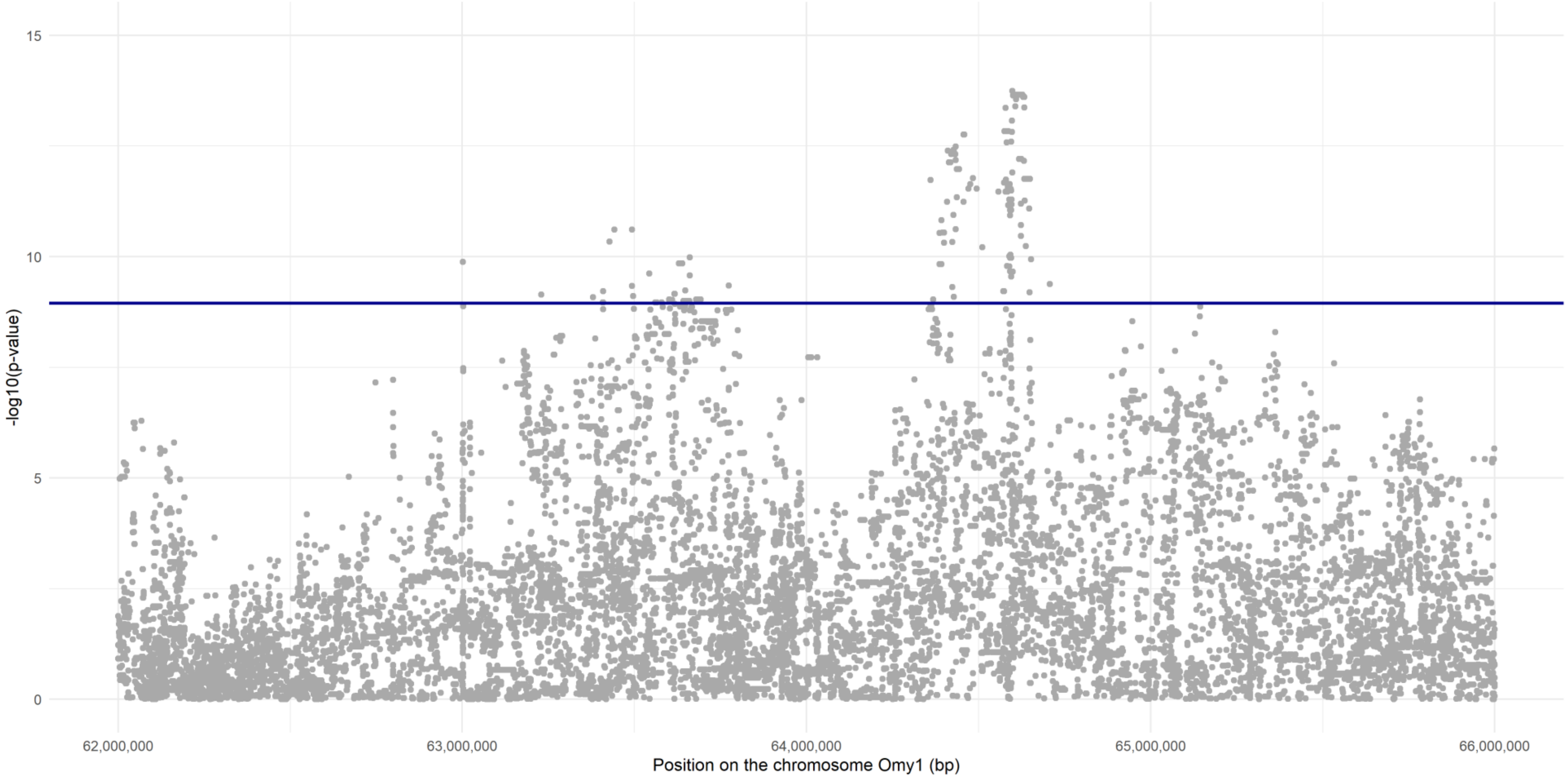

Supplementary Figure S2. Manhattan plot for the GCTA-seq analysis for SNPs located between 62 and 66 Mb in the chromosome Omy1. The dark blue line is the 1% genome wide threshold estimated with Bonferroni correction.

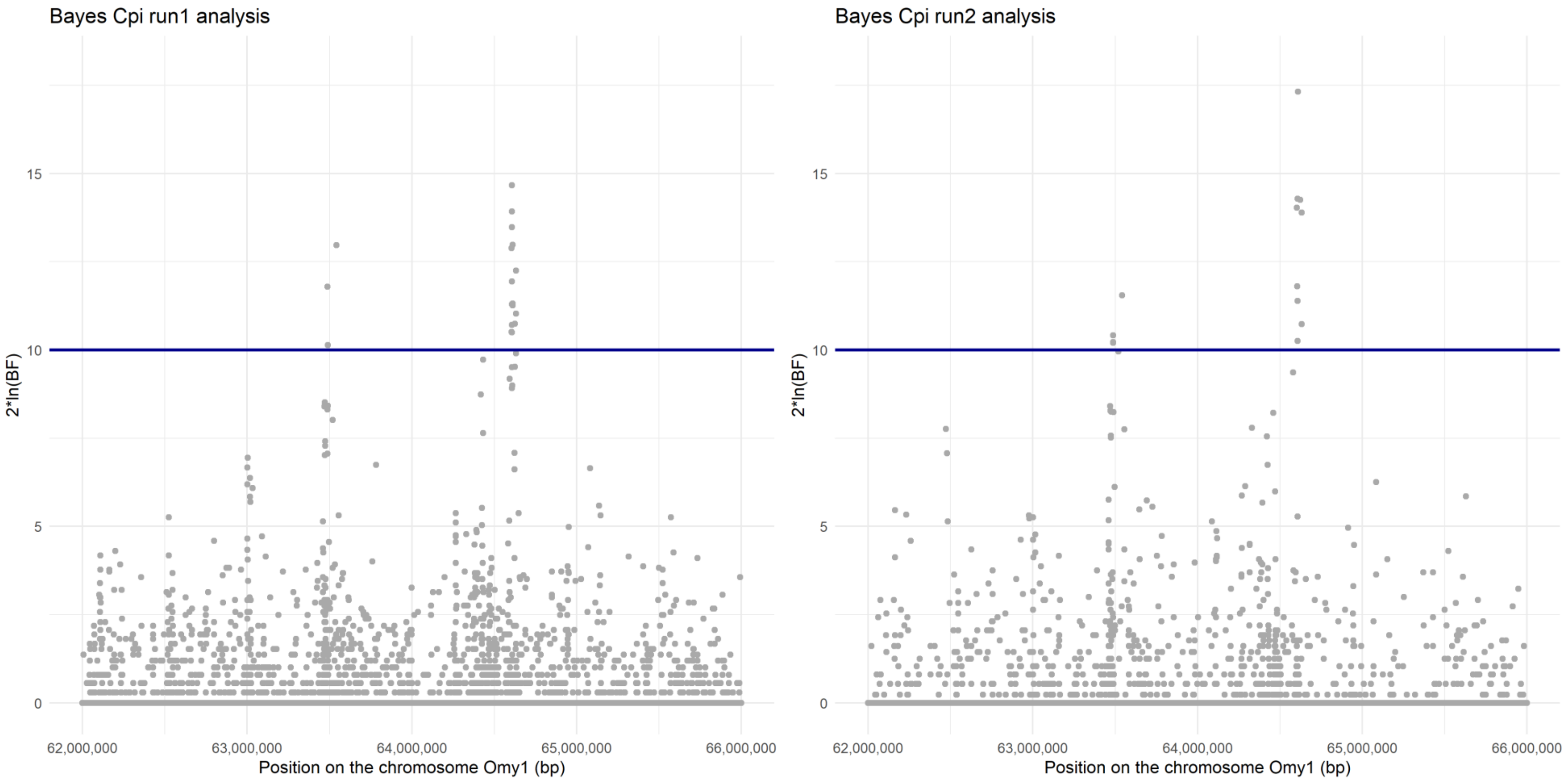

Supplementary Figure S3. Manhattan plots for the two BCπ-seq analysis performed with two seeds.
The dark blue line correspond to the very strong evidence in favour of a QTL threshold. Bayes Cπ run1 and run2 correspond to the outputs of the two seed to initiate the MCMC algorithm. BF = Bayes Factor
